# New EST-SSR Markers for Individual Genotyping of Opium Poppy Cultivars (*Papaver somniferum* L.)

**DOI:** 10.3390/plants9010010

**Published:** 2019-12-19

**Authors:** Jakub Vašek, Daniela Čílová, Martina Melounová, Pavel Svoboda, Pavel Vejl, Radka Štikarová, Luboš Vostrý, Perla Kuchtová, Jaroslava Ovesná

**Affiliations:** 1 Czech University of Life Sciences Prague, FAFNR, Department of Genetics and Breeding, Kamýcká 129, 6 Suchdol, 16500 Prague, Czech Republic; cilova@af.czu.cz (D.Č.); melounova@af.czu.cz (M.M.); vejl@af.czu.cz (P.V.); stikarova@af.czu.cz (R.Š.); vostry@af.czu.cz (L.V.); 2 Crop Research Institute, Division of Crop Genetics and Breeding, Drnovská 507/73, 6 Ruzyně, 16106 Prague, Czech Republic; pavel.svoboda@vurv.cz (P.S.); ovesna@vurv.cz (J.O.); 3 Czech University of Life Sciences, FAFNR, Department of Agroecology and Crop Production, Kamýcká 129, 6 Suchdol, 16500 Prague, Czech Republic; kuchtova@af.czu.cz

**Keywords:** *Papaver somniferum* L., Papaveraceae, EST-SSR, microsatellites, individual genotyping

## Abstract

High-quality simple sequence repeat (SSR) markers are invaluable tools for revealing genetic variability which could be utilized for many purposes, such as breeding new varieties or the identifying current ones, among other applications. Based on the analysis of 3.7 million EST sequences and 15 genomic sequences from bacterial artificial chromosome (BAC) libraries, 200 trinucleotide genic (EST)-SSR and three genomic (gSSR) markers were tested, where 17 of them fulfilled all criteria for quality markers. Moreover, the reproducibility of these new markers was verified by two genetics laboratories, with a mean error rate per allele and per locus equal to 0.17%. These markers were tested on 38 accessions of *Papaver somniferum* and nine accessions of another five species of the *Papaver* and *Argemone* genera. In total, 118 alleles were detected for all accessions (median = 7; three to ten alleles per locus) and 88 alleles (median = 5; three to nine alleles per locus) within *P. somniferum* alone. Multivariate methods and identity analysis revealed high resolution capabilities of the new markers, where all but three pair accessions (41 out of 47) had a unique profile and opium poppy was distinguished from other species.

## 1. Introduction

*Papaver somniferum* L. (opium poppy) is a well-known and extensively studied representative of the *Papaver* genus, numbering over 100 species [1]. As a traditional plant, it has accompanied man since at least the early Neolithic age [2,3]. Today, *P. somniferum* is an economically important plant grown throughout the world for two main reasons. Firstly, *P. somniferum* serves as a source of secondary metabolites such as morphine, thebaine, codeine, or noscapine, which are utilized by pharmaceutical companies for producing medicines with analgesic, antitussive, sedative, or anti-tumor effects [4,5]. Its dark side is infamously the abuse of its sap (“opium latex”) for the production of heroin [6]. Secondly, poppy seeds do not contain a high amount of morphinan alkaloids when they are properly harvested and/or treated [7,8]; thus they are also used in the food industry, typically in Central and Eastern Europe, given their historical and cultural background.

The opium poppy is a diploid (2n = 14) annual plant with a prevailing self-pollinating mode of cross-breeding [9,10]. This characteristic simplifies the whole breeding process, which starts from heterogeneous material of different origin and usually continues by the selection of individual “mother” plants with the required properties. Then follows several generations of self-pollination, necessary for genetic stabilization, homozygotization, and obtaining a sufficient amount of seeds. New varieties should fulfill the UPOV requirements (The International Union for the Protection of New Varieties of Plants) abbreviated DUS (Distinctness, Uniformity, Stability; see more details on UPOV webpages) and could be defined as a pure line, which is characterized by a very high degree of homozygosity and homogeneity. It should be mentioned that other breeding strategies are possible (e.g., creation of hybrid varieties) but are rarely performed.

Modern poppy varieties are classified into three broad categories, as their respective utilization reflects the breeding purpose [11]. The industrial category represents varieties with generally high alkaloid content or varieties producing high amounts of specific chemical compounds like thebaine or noscapine [5]. In contrast, the culinary category includes varieties where the breeding was aimed at low alkaloid content and enhanced flavor. Some varieties combine features of the previously mentioned categories and thus are classified as dual purpose. An extra category could represent varieties for ornamental purposes. Irrespective of category, there is a high demand for suitable markers which allow one to evaluate the diversity among the germplasm collection, accelerate the breeding process, or identify individual varieties. There are typically two approaches, which are quite often intertwined; the first one is based on chemoclassification according to type and number of chemical compounds, and the second one exploits different types of DNA markers.

The application of highly sensitive methods such as gas (GC) or liquid chromatography (HPLC) with mass spectrometry (MS) naturally allows for the discrimination between the *Papaver* species [12], determining high and low alkaloid content varieties [1,13], the geographical origin of poppy varieties [14], or promising breeding lines [15,16]. Nevertheless, it seems that these methods are not sufficiently reliable at the fine scale necessary for the discrimination of individual varieties. This is probably due to the complex influence of many environmental conditions changing the alkaloid content [6,17,18] leading to a great intra-cultivar variability. Here, DNA markers, which are not influenced by these factors, have proven to be helpful. The first attempts to evaluate genetic variability and diversity among varieties were done by random amplified polymorphic DNA (RAPD), inter-simple sequence repeat (ISSR), or amplified fragment length polymorphism (AFLP) methods [19,20,21,22] because these methods do not require a priori knowledge about the genome. Simultaneously, with an accumulation of sequencing data, microsatellite markers were also developed [23,24,25,26]. 

Microsatellites, or simple sequence repeats (SSR), belong to broad family of tandemly repetitive DNA. They are characterized by a short nucleotide motif of 1–6 bp (also called repeat unit) with a total length up to 100 bp [27,28], where their further classification depends on the number of the repeat units, the specific nucleotide motif, the composition of the whole repetition and the localization within the cell or the genome. They have many desirable attributes to make them suitable as molecular markers. They are highly abundant, (nonrandomly) distributed throughout the whole genomes of both prokaryotic and eukaryotic organisms, hypervariable, multiallelic in nature, locus specific, and codominant [29,30]. Moreover, SSR markers are also amenable for multiplexing and high-throughput genotyping [31,32].

When establishing microsatellites as a marker, it is necessary to obtain information about the sequences flanking their own repetitive region. The sequences could be retrieved directly from genomic DNA, cDNA (complementary DNA) or only a short part of cDNA called EST (expressed sequence tag), both of which are created by the reverse transcription of RNA. Thus, one can classify SSR markers as genomic (gSSR) or genic (EST-SSR). Genic SSR markers possess several advantages over genomic SSR markers, as they are considered more reliable and robust, could be potentially directly connected to associated traits because they refer to polymorphism in the expressed portion of their genome and are more transferable across to closely relative species [29,33]. On the other hand, they are usually less polymorphic than their genomic counterparts and their amplicon size can differ from expectation given the presence of introns in flanking regions [29].

The usefulness of SSR markers in the field of plant genetics is proven in their application in the creation of linkage maps, the characterization of genetic diversity in germplasm collections, marker-assisted selection, QTL identification, gene mapping, DNA fingerprinting, paternity analysis, and evolutionary or populations studies, among others [29,32,34,35]. These analyses were made on many plant species, such as *Ambrosia artemisiifolia* [36], *Vigna unguiculata* L. Walp. [37], *Brassica oleracea* [38], *Solanum tuberosum* [39], and *Oryza sativa* L. [40], and demonstrated the versatility of microsatellite markers. The number of SSR markers for the opium poppy is increasing, but they are poorly characterized in most cases; therefore, only a few of them are suitable for variety identification. Thus, our aim was to develop and characterize a new set of polymorphic markers resembling Mendelian inheritance in diploid organisms, amenable for multiplexing and automatic processing of large quantities of samples.

## 2. Results

### 2.1. Bioinformatic Analysis

Searching within ~382,000 cDNA sequences for loci with a trinucleotide motif and 10 or more repeats led to 585 loci being found, where for 308 loci (52.6%) primers were designed and 299 (51.1%) of them were unique according to GMATA software (Genome-wide Microsatellite Analyzing Tool Package). Similar searching within BAC (bacterial artificial chromosome) genomic sequences revealed 12 gSSR loci. Primers were designed for all of them, but subsequent analysis left only three (25%) primers fulfilling the quality requirements.

### 2.2. Marker Testing

As a first step, a total of 200 EST-SSR and three gSSR markers were tested by agarose gel electrophoresis, where 166 (~82%) of them produced a visible band(s). This amplification control was done in two rounds on a subset of eight accessions. The first round of amplification was done at T_a_ = 60 °C and the non-amplifying loci were tested again at T_a_ = 57 °C.

The second step comprised of one (preliminary test with four accessions only) or two (all accessions) rounds of fragment analysis by capillary electrophoresis for 38 promising markers (~18.7%), but only 17 (~8.4%) of them fulfilled all the requirements for high-quality markers. The potential markers were mostly excluded due to multiple peak profiles, suggesting amplification of two or more loci at once, even after several rounds of primer redesign. The results of studies involved in SSR markers development are summarized for comparison purposes in Table 1.

### 2.3. Marker Polymorphism, Transferability, and Reproducibility

The analyzed samples were divided into three groups in order to gain a better understanding of the distribution of genetic variability (Table 2). The first group include all accessions and represents the total variability detected by our microsatellite panel; the second group comprises of the accessions of *P. somniferum*, irrespective of purpose class; and the last group contains only the cultivars of *P. somniferum* amenable for culinary usage, although some of them are classified as dual purpose or even industrial. In total, 118 alleles were detected with a median of seven alleles and three to ten alleles per locus. As expected, the number of alleles in other groups decreased to 88 (*P. somniferum* only) or 77 (*P. somniferum* culinary cultivars) alleles with a median of five and four alleles with three to nine and two to nine alleles per locus, respectively. The observed heterozygosity was generally very low and ranged between 0.048–0.190 with a median of 0.110 for the first group, between 0.026–0.158 with a median of 0.050 for the second group, and between 0.000–0.176 with median 0.060 for the third group (Table 2). We also calculated the PIC (polymorphic information content) to get a different kind of estimation of marker polymorphism. Its values varied between 0.245–0.757 with a median of 0.575 for the first group, between 0.164–0.764 with a median of 0.517 for the second group, and between 0.081–0.779 with median of 0.468 (Table 2).

Cross species transferability was tested on nine accessions of five species of the genus *Papaver* and *Argemone*, both belonging to the Papaveraceae family (Table S1). The highest transferability was reached for *P. nudicaule*, with 13 amplifying markers, followed by *P. orientale* (10), *P. glaucum* (9), *P. rhoeas* (8), and *A. mexicana* (2). Only 1 out of the 17 markers, namely OPEST099, amplified for each tested species and another five (OPEST48c, OPEST53c, OPEST102b, OPEST120b, and OPEST131) amplified for each species but one. On the other hand, only the marker OPEST156 was strictly specific for *P. somniferum* and did not produce any detectable signal for the other species in both labs. However, five out of seventeen markers (29.41%) amplified for all species within the *Papaver* genus, seven markers (41.18%) amplified for all species but one within the *Papaver* genus, 12 markers (70.60%) amplified for two out of four *Papaver* species, 15 markers amplified (88.24%) for at least one species within the *Papaver* genus, and only two markers (11.76%) produced a signal peak across the genera *Papaver* and *Argenome*.

For the *P. somniferum* dataset, the mean *e_a_* (mean error per allele) and *e_l_* (mean error per locus) reached the same value equal to 0.17%. In other species, reproducibility was substantially lower with mean *e_a_* = 27.18% and mean *e_l_* = 32%. The detailed information about the error rate per allele and per locus for the other species dataset is presented in Table S2.

### 2.4. Assessment of Genetic Diversity and Discrimination Power of Markers

**Figure 1:**
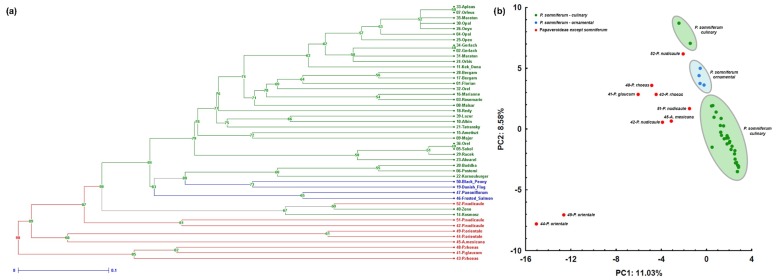
(**a**) Hierarchical cluster analysis (CLU) and (**b**) principal component analysis (PCA) analysis where *P. somniferum* accessions (green) with distinguished ornamental varieties (blue) and other species (red) are shown.

Resolving the similarities/dissimilarities between the accessions was done with the help of two exploratory methods, namely hierarchical cluster analysis (CLU) and principal component analysis (PCA). As the best clustering algorithm, UPGMA (unweighted pair group method with arithmetic mean) was selected according to the highest value of CC (cophenetic correlation coefficient) and lowest *delta* parameter (Table S3). As can be seen in Figure 1a,b, the new SSR markers were able discriminate *P. somniferum* accessions (green, blue color) from other species (red color) with the exception of one *P. nudicaule* sample. Moreover, within *P. somniferum* accessions, the ornamental varieties (blue color) were found to be most similar to each other, although they did not create one enclosed cluster. Furthermore, according to CLU (Figure 1a) it is visible that new markers discriminate all but two pairs of accessions—that is, Aplaus vs Orfeus and Orel vs Sokol (all *P. somniferum* varieties). There is another pair of accessions (Gerlach-2 and Gerlach-34) with no differences, but these accessions belong to the same variety. Interestingly, the same situation was not observed for the Bergam, Maraton, Opal, or Orel varieties (Figure 1a).

A similar result was obtained by PCA analysis, where the projection onto two principal axes is shown in Figure 1b. Two synthetic variables explained 19.61% of the total amount of variability, where the first axis divided *P. somniferum* accessions from the other species and the second axis further separated accessions both within *P. somniferum* and the other species. We also observed an analogous result during CLU for one *P. nudicaule* sample, showing high similarity to some *P. somniferum* accessions, and the existence of two *P. somniferum* “outliers” represented by the Kozmosz and Zeno varieties.

Identity analysis revealed that an exact match in all tested loci was found for the same pairs of accessions as mentioned for CLU analysis (Table S4). It also means that 41 unique profiles out of 47 accessions were found. When allelic mismatch starting from one to five alleles was allowed, the number of same pairs of accessions rose to 9, 15, 25, 37, and 52, respectively (Tables S4–S9a,b).

## 3. Discussion

Data mining allows for the fast designing of tens to hundreds of thousands of SSR markers in silico, but their quality and usefulness can only be proven by empirical analysis in the “wet” lab. We tried to raise our chances of obtaining a set of quality markers with sufficient polymorphism and potentially single locus specificity by setting up several criteria. At the very beginning, we limited our search to only microsatellites with a trinucleotide motif and no less than 10 repeats. Besides SSRs with a trinucleotide motif, we could also use hexanucleotide microsatellites, but microsatellite abundance with longer motifs typically decreases within the genome [41,42], and information about their polymorphism is lacking in the literature. The reason for using microsatellites with a motif of a multiple of three is obvious, because potential mutations preserving the reading frame are less severe than frameshift mutations. Moreover, it is well known that microsatellite polymorphism increases with total length, due to the higher chance of slippage of DNA polymerase during replication [30,43].

As we found, the second goal (single locus specificity) imposed a limit on the markers of choice. *P. somniferum* has a complex, not yet fully understood evolutionary history, probably including genomic introgression of two or even three species [9]. Thus, *P. somniferum* (2n = 22) could be classified as an alloaneuploid, where its basic chromosome number x = 11 is derived from the more ancestral x = 7, given the existence of a hypothetical triploid hybrid that may have preceded the speciation of *P. somniferum* [9]. Although Marciano et al. [6] mentioned that the diploid nature of *P. somniferum* simplified the workflow for genetic identification and compared it with the development of markers for human identification, we worried that they probably underestimated the evolutionary complexity of the genome development typical for flowering plants and the consequences of allopolyploidy [44,45]. Namely, for *P. somniferum*, analysis of a 401 kb long genomic sequence of gene cluster for noscapine synthesis was performed, revealing gene duplicities, structural rearrangement, and many DNA or retrotransposable elements [5]. This implies that similar structures should not be limited to only one genomic region and that analogous situations could occur throughout the whole poppy genome. Despite such unfavorable conditions, our goal was to develop markers resembling typical Mendelian inheritance in a diploid organism.

The empirical verification of locus specificity was ensured by two complementary approaches. The first idea was that most of our accessions were varieties and, by definition, should fulfil the DUS criterion. Thus, for varieties of autogamous plants, like *P. somniferum* is, we expected a very high degree of homozygosity, i.e., a low heterozygosity (Table 2). This was verified after the analysis of all the final markers, where 24 out of 34 (~71%) *P. somniferum* varieties (including the varieties for ornamental purposes) were homozygotes for all markers and most of the rest (six out of ten) were heterozygotes for only one or two markers (i.e., homozygotes for 15 or 16 markers). It helped to eliminate markers where a clear profile and a maximum of two peaks per sample were detected, but all or almost all plants seemed to be heterozygotes, which was improbable given our knowledge about the tested material. Only Lee et al. [26] also tried to exclude nonspecific markers by the elimination of markers with three or more peaks per sample, but they were unable to recognize and discard seemingly locus-specific markers with a heterozygous-like profile because they tested unknown genetic material obtained through narcotic seizures. The second idea relied on the verification of locus specificity through the direct sequencing of PCR products, followed by comparison against reference sequences obtained during data mining, where only sequences with little or no noise were accepted. This noise in several markers suggested weak co-amplification from other locus/loci, but the low amount of such sequences was not detectable, even with highly sensitive capillary electrophoresis, and thus this seems to be unimportant for methods based on fragment analysis.

At the beginning of marker testing, we obtained the same percentage of successful amplification (82%) as Selale et al. [25], but the number of retained markers was lower. Selale et al. [25] tested 93 EST-SSR markers and retained 67 markers (72%), versus 203 tested markers and 17 (8.4%) retained markers in this study (Table 1). Moreover, when we take into account the total number of tested primer pairs (272) for 203 markers, then the percentage of retained markers drops to 6.25%, a ratio of 1:15 (accepted: discarded markers). Celik et al. [24] also reported a higher yield of markers in opium poppies when they tested 100 genomic SSR and 53 (53%) were found to be useful (Table 1). Such discrepancy is probably caused by a subtle yet important difference in the methodology of the mentioned studies. They analyzed mixtures of 10 or 20 plants per sample and each allele was counted by the presence (1) or absence (0) of a peak signal of appropriate size, with no restriction on how many peaks (i.e., alleles) were allowed per marker. Such an approach could be very useful when one wants to quickly retrieve many markers, obtain a relatively large amount of data on a genome-wide level, and overcome the problem with polyploidy, but everything comes at a price. There is a loss of the codominant nature of the SSR markers and the information about the source of polymorphism. This could lead to bias in the analyses where a higher level of precision is needed (e.g., mapping or individual genotyping). This approach is also technically challenging for routine screening because such markers are not easily amenable for multiplexing without an elevated risk of error, given the higher order of interactions between the primers with unknown specificity, and the individual analysis of tens of such markers is costly and time consuming.

A total of 17 selected markers identified 118 alleles for all accessions, with a median of seven alleles per locus, which is comparable even with the variability of the genomic SSR markers in populations of the allogamous species *P. rhoeas* [46]. Nonetheless, variability within *P. somniferum* itself is more important for our purpose, where the number of alleles decreased by about 25% (88 alleles, median = 5) for all *P. somniferum* accessions and about 35% when we take account only culinary varieties (77 alleles, median = 4). Unfortunately, the direct comparison of variability within *P. somniferum* species is only possible with the study of Lee et al. [26], where they found at six loci 17 alleles with a mean of 2.83 alleles per locus, and Mičianová et al. [23] who reported 20 alleles at 8 loci with a mean of 2.5 alleles per locus (Table 1). More extensive studies [24,25] with a similar number of samples adopted a different strategy of marker development and analysis (see the previous part of discussion) and we can only perform an indirect and thus biased comparison when we accept the premise that one fragment is equal to one allele. In the former study [24], 207 (all accessions) and 90 (*P. somniferum* accessions) fragments per 53 gSSR markers were detected, with a mean of 3.9 and 1.7 fragments per marker, respectively. The latter study [25] reported 562 (all accessions) and 253 (*P. somniferum*) fragments per 67 EST-SSR markers, with a mean of 8.4 and 3.77 fragments per markers, respectively (Table 1). We also performed an empirical evaluation of 21 published markers (Table S10) where all but two (psom 12 and psom 17) of the markers were discarded due to multiple peak profiles or insufficient polymorphism (our internal limit was at least 3 alleles per marker for *P. somniferum* accessions) and thus we were unable to compare it with our SSR panel. All this information showed the same or higher levels of performance in newly developed markers and a congruence about relatively low genetic variability within varieties of *P. somniferum*.

Cross-species transferability of new markers within the genus seems to be rather low (29.41%) when we accept the published range from 23% to 96% [33], and this probably depends on the sequence conservation of the involved genes given their functional importance and the phylogenetic distances of compared species [29]. Surprisingly, a much higher level of transferability within the *Papaver* genus was reported not only for the EST-SSR markers (97%) [25], but also for genomic SSR (at least 88.7%) [24]. It seems that these markers targeted more conservative regions of the genome, both inter and intragenic, or lacked specificity. In contrast, Lee et al. [26] mentioned two out of six markers (33%) successfully amplifying for at least one species of the *Papaver* and *Escholzia* genera, and one marker (16.67%) amplifying both for the genera *Papaver* and *Escholzia*, which is closer to our results. Although the number of amplifying markers in other species is lower, they still represent a valuable source of markers for the less studied *Papaver* species, where a sufficient amount of sequences and markers is still lacking.

As far as we know, this is the first study reporting marker reproducibility within or between papaver species and thus we are unable to directly compare our results with other articles dealing with *P. somniferum* species. It seems that our mean error rate per locus (in our case *e_l_* = *e_a_* = 0–2.86%, mean = 0.17%) is lower than the usual mean error rate, which ranges from 0.5% to 1% [47]. This result is also in concordance with newer studies in olive (*Olea europaea*) where *e_a_* ranged from 0.7% to 6.2% with a mean of 2.26% [48] or *Alnus incana* (*e_a_* = 0–8%, mean = 1.4%) and *Alnus glutinosa* (*e_a_* = 0–6.3%, mean = 1.3%) [49]. Unfortunately, none of these studies mentioned *e_l_*.

A substantially different situation was revealed by comparison with other species’ datasets, where all metrics showed a very high error rate (Table S2). The main reason probably lies in the evolutionary distances of the assessed species, causing a lower specificity and thus reduces the amplification capability of markers primarily developed for *P. somniferum*. This corresponds with the most frequent source of error, which was amplification failure. Other explanations include human factors, cross-contamination, or different criteria for accepting a signal as an allele; but these kinds of problems might not be restricted to other species datasets, and it was not noticed for *P. somniferum* datasets. It is also necessary take into account that few samples per marker were evaluated and any mismatch led to a high error rate. As a result of these findings, we advise any usage of our markers in species other than *P. somniferum* to be taken with caution. This is also the reason why a more conservative approach was chosen and a lower transferability was reported, because only markers amplifying in both labs are labelled by a + sign (Table S1).

The new microsatellite system distinguished all accessions except for two pairs of varieties (Orel vs Sokol and Aplaus vs Orfeus), as proved by CLU and PCA analyses (Figure 1a,b). Unfortunately, little is known about the breeding history or pedigree of the mentioned cultivars, but both Orel and Sokol are white-seeded poppies. Furthermore, there is information available which shows that Orel and another white-seeded variety, Racek, were selected as individual plants from an unstable population of a local (unnamed) variety [50], so they should share some genetic similarities (Figure 1a). These cultivars have the same breeder (Oseva Pro Ltd., Opava, Czech Republic), thus we can speculate that Sokol also has its origin in the same local variety; we can then talk about isolines, which could be difficult to resolve, even with additional markers. The second pair of cultivars belong to a category of blue-seeded poppies, but they have different breeders, and no information about the breeding history of Aplaus has been published. This result suggests that the gene pool actually used for new varieties is quite narrow and/or that our system lacks robustness, as it was implicated by the identity analysis (ID) paired comparison of varieties. Although we could argue that the probability of a random match is much lower (e.g., pID = 1.09 × 10^−16^; Table S4) than the probability (pID = 1.04 × 10^−3^) published by Mičianová et al. [23], one should treat such values carefully and also look behind the numbers to see their meaning. Such estimators were developed for application in forensic genetics or studies of wild populations and calculate the probability of a random match within an idealized population according to the Hardy–Weinberg law and other assumptions (see more about the topic in [51,52]). In contrast, our study and others analyzed varieties bred by man and they cannot be considered as a population of randomly mating individuals by any means and many varieties could share a substantial portion of their genomes due to their origin, breeding history, and pedigree. This is the reason why we disagree with the argumentation in [23] and, despite the very low p-values for a random match in our case, we regard such values as uninformative and, thus, the obtained result leads us to have doubts about the system’s robustness and to call for a higher number of markers. The last interesting outcome from multivariate analysis showed that there were differences between accessions of the same variety, such as Bergam, Maraton, Opal, or Orel (Figure 1a). At the present time, it is not possible to conclude what the source of these differences is, but probable explanations include some degree of internal variability within varieties, human error (e.g., wrong label, unintended crossing in gene bank), or various degrees of seed stock influencing the purity of the seeds (C1 vs E degree).

## 4. Materials and Methods 

### 4.1. Plant Material

In total, 29 varieties of *Papaver somniferum* (opium poppy), which included all three types of variety classified according to purpose (culinary, dual purpose, pharmaceutical/industrial), 4 varieties of *P. somniferum* typically used for ornamental purpose, 4 other species of the genus *Papaver* (*P*. *glaucum*, *P. nudicaule*, *P*. *orientale*, and *P. rhoeas*) and 1 distant relative species of the genus *Argemone* (*Argemonemexicana*), were collected. In total, 47 accessions were analyzed, because the same varieties from multiple sources were received in several cases (Table 3). All accessions were obtained in the form of seeds. These seeds were put into separated flowerpots and planted in a greenhouse under regulated conditions. Later, a few leaves of each plant were cut in the plant’s phenological phase 13–15 according to the BBCH (Biologische Bundesanstalt, Bundessortenamt und Chemische Industrie) scale [53] and were utilized as source material for the isolation of DNA. Thus, each sample contained DNA from one plant.

DNA isolation was done by the DNeasy Plant Mini Kit (Qiagen, Hilden, Germany) according to the manufacturer’s recommendations. The quality and quantity of the isolated DNA was verified by UV spectrometry (S-111107AW nanophotometer, Implen) and electrophoretic separation in 1% (*w*/*v*) agarose gel.

### 4.2. Bioinformatic Analysis and Primers Design

Filtered and clipped cDNA sequences were downloaded from the Sequence Read Archive (SRA) of the National Center for Biotechnology Information (NCBI). Only the sequences produced by pyrosequencing were chosen, because 454 sequencing typically produces longer reads than Illumina and thus there is a higher chance of obtaining a sufficiently long stretch of simple repeats. About 3.7 million cDNA sequences, with a length of 1.89 Gb in total, and 15 genomic sequences from the BAC library, with a total length 2.7 Mb, were analyzed. This was followed by several rounds of data processing and assembling with the help of the online bioinformatics tool EGassembler [54], resulting in 382531 so-called contigs and singletons, with a total length 187 Mb.

SSR mining was done by GMATA software [55], where microsatellites with a trinucleotide motif and 10 or more repeats were utilized for marker development. The same software was used for the preliminary primer design for amplicons within the range 100–300 bp and Tm 60 °C. When necessary, other primers were designed using Primer3 plus [56,57] and OligoEvaluator software (an online web tool provided by Sigma-Aldrich, St. Luis, MO, USA).

Moreover, 21 genomic SSR (gSSR) or EST-SSR markers (Table S10) published by Celik et al. [24] and Lee et al. [26] for *P. somniferum* and Kati et al. [46] for *P. rhoeas* were chosen and analyzed for comparison purposes.

### 4.3. SSR Loci Amplification

A PCR reaction mixture, with a total volume of 10 µl, included 10 ng of DNA, 0.2 µM F and R primer (Table 4, Tables S11 and S12), and 1× Multiplex PCR Master Mix Plus (Qiagen, Hilden, Germany). The temperature profile was as follows: 1 cycle of predenaturation at 95 °C for 600 s, 35 cycles of denaturation at 94 °C for 30 s, annealing at 57 °C or 60 °C for 90 s and elongation at 72 °C for 60 s, and 1 cycle of final elongation at 60 °C for either 600 s (for agarose gel electrophoresis) or 4800 s (for capillary electrophoresis). The amplification was done by a C-1000 (Bio-Rad, Hercules, CA, USA) or T-gradient Thermo (Analytic Jena, Jena, Germany) thermocycler. The presence of amplicons and the expected size control was done by electrophoretic separation in 2% (*w*/*v*) agarose gel with a size standard GeneRuler 100 bp DNA Ladder (Thermo Fisher Scientific, Waltham, MA, USA). Only the amplicons with one band and in the expected size range were chosen for the next step of analysis by capillary electrophoresis. There were also several cases when one band was observed but the fragment size was two to eight times higher than expected. Such fragments were sequenced, and new primers were eventually designed prior to capillary electrophoresis.

### 4.4. Fragment Analysis

The chosen markers were analyzed by capillary electrophoresis and thus F primer was fluorescently labelled by 6FAM, VIC, NED or PET dye. The amplification condition and temperature profile of PCR was the same as for agarose gel electrophoresis. The verification of the results and the reproducibility control was ensured by analysis of all accessions, both in the molecular laboratory of the Department of Genetics and Breeding (DGB) and in the molecular laboratory of the Crop Research Institute (CRI) on different capillary instruments. In the former laboratory, PCR products were diluted with deionized water at a ratio of 1:99–199 depending on tested marker and 1 µl of diluted PCR product was mixed with 12 µL Hi-Di formamide (Thermo Fisher Scientific, Waltham, MA, USA) and 0.2 µl of GeneScan LIZ600 size standard (Thermo Fisher Scientific, Waltham, MA, USA). The samples were separated by ABI PRISM 310 (Thermo Fisher Scientific, Waltham, MA, USA) with a 47 cm-long capillary filled with POP4 polymer (Thermo Fisher Scientific, Waltham, USA). For fragment data analysis and allele identification, the GeneMapper v4.1 program (Thermo Fisher Scientific, Waltham, MA, USA) was utilized. In the latter laboratory, the PCR products were diluted with deionized water in a ratio of 1:65–199 and 1 µL of appropriate product was mixed with 10 µL of Hi-Di formamide (Thermo Fisher Scientific, Waltham, MA, USA) and 0.2 µL of GeneScan LIZ500 size standard (Thermo Fisher Scientific, Waltham, MA, USA). Separation was done by ABI 3500 (Thermo Fisher Scientific, Waltham, USA) with a 61 cm-long capillary filled with POP7 polymer (Thermo Fisher Scientific, Waltham, MA, USA). Blind analysis irrespective of the DGB lab results was done in GeneMapper v5.0 (Thermo Fisher Scientific, Waltham, MA, USA).

Due to the different conditions in both labs, which influenced fragment mobility and thus allele size estimation, the allelic size standards were developed, and the nomenclature defined for each allele in the DGB lab. Allelic ladders (ALs) containing all or almost all of the detected alleles depending on the marker were created by mixing the PCR products of the appropriate genotypes with a ratio of 2:1 of heterozygotes and homozygotes (see example ladder in Figure S1). The pooled PCR products were further diluted in a ratio of 1:99–350 with deionized water and 1 µL of appropriate ALs was prepared and separated simultaneously with the tested samples under the same conditions as mentioned earlier.

### 4.5. Sequencing

The locus specificity of each marker tested by capillary electrophoresis was checked by the Sanger sequencing method. All markers were amplified in multiple copies, separated in 1% (*w*/*v*) agarose gel, and excised by clean scalpel after 1 h separation. DNA purification was performed using the GenJet Gel Extraction kit (Thermo Fisher Scientific, Waltham, MA, USA) according to the manual’s recommendations. The clean amplicons, together with the specific primers, were sent to the Eurofins Genomics (Eurofins Genomics Germany GmbH, Ebersberg, Germany) sequencing service and were bidirectionally sequenced. The succeeding quality evaluation, assembly, alignment with reference sequence, and necessary manual correction of all sequences was done by Sequencing Analysis Software v5.2 (Thermo Fisher Scientific, Waltham, MA, USA), BioEdit Software v7.0.9.0 [58] and an online version of MUSCLE software [59] available on webpages of The European Bioinformatics Institute (EMBL-EBI).

### 4.6. Statistical Data Analysis

Descriptive statistics, including the number of alleles with frequencies estimation and identity analysis (ID), was performed in Cervus v3.0.3 software [60]. ID was done by a series of paired comparisons of each accession with a 0–5 allowed allele mismatch. STATISTICA software v13.3 [61] was utilized for exploratory data analysis (EDA) by principal component analysis (PCA) and DARwin5 software [62] for EDA by the hierarchical clustering (CLU) method. For the purpose of CLU analysis, the microsatellite data were transformed to binary data with presence (1) and absence (0) coding for alleles. The created distance matrix was based on the Dice coefficient [63] with 10,000 bootstrap steps. The best clustering method was selected according to the highest value of the cophenetic correlation coefficient (CC) and lowest *delta* parameter in the NCSS program [64]. As an input for PCA analysis, a trinary matrix of individual allelic frequencies was created, where the frequency had a value of 1 or 0 for presence or absence of the appropriate allele for homozygotes, whereas heterozygotes had an allele presence value of 0.5.

For the reproducibility analysis, two metrics quantifying error rates were utilized—the error rate per allele (*e_a_*) and the error rate per locus (*e_l_*) according to Pompanon et al. [47]. Each error rate was calculated as the number of mismatches between reference genotypes (here DGB dataset) and replicates (here CRI dataset) where n genotypes had been genotyped t times. The analysis was done separately for *P. somniferum* and other species.

The mean error rate per allele (*e_a_*) is expressed as a ratio between the number of allelic mismatches (*m_a_*) and the number of replicated alleles (*2nt* for diploid organisms) [47].

*e_a_* = *m_a_*/2*nt* (1)

The mean error rate per locus (*e_l_*) is expressed as a ratio between the single-locus genotypes, including at least one allelic mismatch (*m_l_*), and the number of replicated single-locus genotypes (*nt*) [47].

*e_l_* = *m_l_*/*nt* (2)

## Figures and Tables

**Table 1:** Comparison of studies reporting the development of simple sequence repeat (SSR) markers in opium poppy.

Nr. of Tested Markers	Amplifying Markers	Useful Markers	Total Nr. of Alleles*/Fragments**	Mean per Locus (Papaveroideae)	Mean per Locus (*P. somniferum*)	Transferability ^a^ (%)	Study
100	96	53	207 **	3.9	1.7	97	[24]
93	76	67	562 **	8.4	3.77	88.7	[25]
22	17	6	17 *	-	2.83	33	[26]
12	12	8	20 *	-	2.5	-	[23]
203	166	17	118 *	6.94	5.18	29.41	this study

^a^ cross-species transferability.

**Table 2:** Number of alleles per locus (k), observed heterozygosity (Ho) and polymorphic information content (PIC) for all three groups of accessions.

Marker	All Accessions			*P. somniferum*			*P. somniferum*-Culinary		
	k	H_O_	PIC	k	H_O_	PIC	k	H_O_	PIC
OPEST026	10	0.190	0.724	8	0.158	0.682	7	0.176	0.662
OPEST048c	9	0.114	0.599	7	0.053	0.536	6	0.029	0.450
OPEST051c	5	0.077	0.538	5	0.079	0.517	5	0.088	0.485
OPEST053c	10	0.109	0.674	6	0.053	0.598	6	0.059	0.551
OPEST061	7	0.119	0.694	6	0.132	0.672	6	0.118	0.654
OPEST081c	8	0.190	0.559	6	0.158	0.526	5	0.147	0.503
OPEST086d	3	0.068	0.245	3	0.026	0.164	2	0.029	0.081
OPEST099	8	0.109	0.590	3	0.053	0.428	3	0.029	0.425
OPEST102b	9	0.087	0.505	5	0.053	0.371	4	0.029	0.344
OPEST106	6	0.093	0.703	4	0.079	0.631	4	0.059	0.615
OPEST120b	7	0.159	0.605	3	0.105	0.478	3	0.088	0.432
OPEST126b	6	0.049	0.501	5	0.053	0.422	3	0.029	0.381
OPEST131	6	0.111	0.518	4	0.105	0.399	4	0.088	0.418
OPEST156	3	0.053	0.366	3	0.053	0.366	3	0.029	0.368
OPEST169	6	0.075	0.531	5	0.079	0.517	2	0.088	0.485
OPEST177b	6	0.048	0.349	6	0.053	0.228	5	0.000	0.105
OPGSSR001	9	0.154	0.693	9	0.158	0.699	9	0.088	0.654
mean	6.94	0.104	0.564	5.18	0.084	0.500	4.53	0.068	0.466
median	7	0.101	0.575	5	0.066	0.517	4	0.059	0.468

**Table 3:** Analyzed accessions of *P. somniferum* (opium poppy) and related species.

Accession Number	Assigned Code	Species	Genebank Evidence Number	Variety	Source
1	FLO	*P. somniferum*	15O0800171	Florian	genebank Opava ^a^
2	GER	*P. somniferum*	15O0800148	Gerlach	genebank Opava ^a^
3	ROS	*P. somniferum*	15O0800164	Rosemarie	genebank Opava ^a^
4	OPA	*P. somniferum*	15O0800169	Opal	genebank Opava ^a^
5	SOK	*P. somniferum*	15O0800179	Sokol	genebank Opava ^a^
6	POS	*P. somniferum*	15O0800195	Postomi	genebank Opava ^a^
7	ORF	*P. somniferum*	15O0800190	Orfeus	genebank Opava ^a^
8	MAL	*P. somniferum*	15O0800183	Malsar	genebank Opava ^a^
9	MAJ	*P. somniferum*	15O0800182	Major	genebank Opava ^a^
10	ALB	*P. somniferum*	15O0800159	Albín	genebank Opava ^a^
11	KEK	*P. somniferum*	15O0800093	Kék Duna	genebank Opava ^a^
14	KOS	*P. somniferum*	15O0800173	Kozmosz	genebank Opava ^a^
15	AME	*P. somniferum*	15O0800198	Ametiszt	genebank Opava ^a^
16	MAE	*P. somniferum*	15O0800031	Marianne	genebank Opava ^a^
17	BER	*P. somniferum*	15O0800184	Bergam	genebank Opava ^a^
18	RED	*P. somniferum*	15O0800189	Redy	genebank Opava ^a^
19	DAN	*P. somniferum*		Danish Flag	market
20	BUD	*P. somniferum*	15O0800185	Buddha	genebank Opava ^a^
21	TAT	*P. somniferum*		Tatranský	Červený Újezd ^b^
22	KOR	*P. somniferum*		Korneuburger	Červený Újezd ^b^
23	AKV	*P. somniferum*		Akvarel	Červený Újezd ^b^
24	ORB_II	*P. somniferum*		Orbis	CISTA ^c^
25	OPE_II	*P. somniferum*		Opex	CISTA ^c^
26	ONY_II	*P. somniferum*		Onyx	CISTA ^c^
28	BER_II	*P. somniferum*		Bergam	CISTA ^c^
29	RAC_II	*P. somniferum*		Racek	CISTA ^c^
30	OPA_II	*P. somniferum*		Opal	CISTA ^c^
31	MAR	*P. somniferum*	15O0800181	Maraton	genebank Opava ^a^
32	ORE	*P. somniferum*	15O0800187	Orel	genebank Opava ^a^
33	APL_II	*P. somniferum*		Aplaus	CISTA ^c^
34	GER_II	*P. somniferum*		Gerlach	CISTA ^c^
35	MAR_II	*P. somniferum*		Maraton	CISTA ^c^
36	ORE_II	*P. somniferum*		Orel	CISTA ^c^
39	LAZ	*P. somniferum*		Lazur	Červený Újezd ^b^
40	ZEN	*P. somniferum*		Zeno	Červený Újezd ^b^
41	GLA	*P. glaucum*		seed mixture	market
42	GNO	*P. nudicaule*		Gnome	market
43	RHO	*P. rhoeas*		seed mixture	market
44	PIZ	*P. orientale*		Pizziato	market
45	MEX	*A. mexicana*		seed mixture	market
46	FRO	*P. somniferum*		Frosted Salmon	market
47	PAE	*P. somniferum*		Paeoniflorum	market
48	DAW	*P. rhoeas*		Dawn Chorus	market
49	SCHA	*P. orientale*		Scharlach	market
50	PEO	*P. somniferum*		Black Peony	market
51	NUD_I	*P. nudicaule*		seed mixture	market
52	NUD_II	*P. nudicaule*		seed mixture	market

^a^ part of Oseva Ltd company and Plant oil research institute, ^b^ research station Červený Újezd, ^c^ Central Institute for Supervising and Testing in Agriculture.

**Table 4:** Information about primers, fluorescent dyes, and size range of new genic (EST)-SSR markers.

Marker	Dye		Primer sequence 5′- 3′	Size range [bp]	GenBank AC Number
OPEST026	6FAM	F-	GTGAGGAGGACGAGCTTTTG	105–148	MK744101
		R-	gtttcttCCGTTGTAAAATACCGACTGC		
OPEST048c	PET	F-	CGTGAGAAGCTAGAACAGAAAGA	174–204	MK744102
		R-	TCGTTCACTGAGTTCTGATATGA		
OPEST051c	VIC	F-	GGGTTCTTTTGTTCTACTTCTTTCTT	147–162	MK744103
		R-	AAGGTGTCGGTGCCCAGC		
OPEST053c	NED	F-	TCAATACCCACAAAAGGAGGA	172–203	MK744104
		R-	gtttcttTCAAGACAAAGAAACCAAGCCA		
OPEST061	6FAM	F-	GGCTGCTGCTTCTTTTCATC	191–236	MK744105
		R-	ATAGGGCAAACTGCCTGCTA		
OPEST081c	PET	F-	AGTAAAACGATCCGTACCTACCTGA	133–176	MK744106
		R-	CGTTTTTCTACAGGGTTGATTTCTGA		
OPEST086d	PET	F-	ACCTTTCCCCCTCTTCAGTAGC	223–244	MK744107
		R-	TCCAGTCCACATCAGGATCA		
OPEST099	6FAM	F-	TTAACAGATCCGCATTTCCA	262–288	MK744108
		R-	CACCGATTGTACCACGAAGA		
OPEST102b	VIC	F-	CGCCACCACATATTTCTCTG	184–206	MK744109
		R-	GGTTGTCGGCATAGAAGGAA		
OPEST106	6FAM	F-	CACCAAATCTCATTGCCTGA	166–191	MK744110
		R-	CCCTAATCGGATGGATCAAA		
OPEST120b	6FAM	F-	TAGTGGTTGCTCGTAGCGTC	138–156	MK744111
		R-	TCACGGTTCTTCTATCATGGTG		
OPEST126b	6FAM	F-	GTTTCTCACGGAGGGATTTG	206–228	MK744112
		R-	CCGTTTCCCAACTTCGTAGA		
OPEST131	VIC	F-	GTTCCAAACCACCAACCACA	224–250	MK744113
		R-	TTGTGAGGCCCTAGAGAGGA		
OPEST156	6FAM	F-	TTTAGCTTACAATGGTGGGAGA	264–270	MK744114
		R-	GAAACCGTAGCCAGGTGAAA		
OPEST169	VIC	F-	TCCAACGCAAGCAATTACAA	165–205	MK744115
		R-	GCCACTTCGTAACCCAGGTA		
OPEST177b	VIC	F-	TCTCCGTAACCTGAAGAACAGA	96–112	MK744116
		R-	TGGTGGCAGTGAATTTGAT		
OPGSSR001	VIC	F-	TGCGGCTTCTAATCATCCTT	218–244	MK744117
		R-	CCATCAACTTCGCACAGCTA		

## Supplementary Materials

The following are available online at https://www.mdpi.com/2223-7747/9/1/10/s1, Figure S1: Example of allelic ladder for marker OPEST48c, Table S1: Transferability of EST-SSR markers among other species of Papaveraceae family, Table S2: Error rate per allele and per locus for other species datasets, Table S3: CC and delta values for each tested clustering method, Table S4: Identity analysis with 0 allelic mismatches allowed, Table S5: Identity analysis with one allelic mismatch allowed, Table S6: Identity analysis with two allelic mismatches allowed, Table S7: Identity analysis with three allelic mismatches allowed, Table S8a: Identity analysis with four allelic mismatches allowed, Table S8b: Identity analysis with four allelic mismatches allowed, Table S9a: Identity analysis with five allelic mismatches allowed, Table S9b: Identity analysis with five allelic mismatches allowed, Table S10: Empirical evaluation of 21 published SSR markers, Table S11: Tested EST-SSR markers, Table S12: Tested gSSR markers.
