# Supplementary material for: New EST-SSR Markers for Individual Genotyping of Opium Poppy Cultivars (Papaver somniferum L.)
Source: Plants (Basel). 2019 Dec 19;9(1):10. doi: 10.3390/plants9010010 (PMC7020189; doi:10.3390/plants9010010)
Supplement: Supplementary file 1 [file plants-09-00010-s001.zip › plants-643729-supp/02-Supplement-Plants-643729.docx]

**New EST-SSR markers for individual genotyping of opium poppy cultivars (*Papaver somniferum* L*.*)**

Jakub Vašek^a*^, Daniela Čílová^a^, Martina Melounová^a^, Pavel Svoboda^c^, Pavel Vejl^a^, Radka Štikarová^a^, Luboš Vostrý^a^, Perla Kuchtová^b^, Jaroslava Ovesná^c^

^a^Czech University of Life Sciences Prague, FAFNR, Department of Genetics and Breeding, Kamýcká 129, Prague 6 – Suchdol, 16500, Czech Republic

^b^Czech University of Life Sciences Prague, FAFNR, Department of Agroecology and Crop Production, Kamýcká 129, Prague 6 – Suchdol, 16500, Czech Republic

^c^Crop Research Institute, Division of Crop Genetics and Breeding, Drnovská 507/73, Prague 6 – Ruzyně, 16106, Czech Republic

*Corresponding author

e-mail: [vasek@af.czu.cz](mailto:vasek@af.czu.cz)

phone: +420 22438 2562

**Supplement material**

**Table S1.** Transferability of EST-SSR markers among other species of Papaveraceae family.

| **marker** | *P.glaucum* | *P.nudicaule* | *P.rhoeas* | *P.orientale* | *A.mexicana* |
| --- | --- | --- | --- | --- | --- |
| OPEST026 | - | + | - | + | - |
| OPEST048c | + | + | + | + | - |
| OPEST051c | - | + | - | - | - |
| OPEST053c | + | + | + | + | - |
| OPEST061 | - | + | - | + | - |
| OPEST081c | - | + | - | + | - |
| OPEST086d | - | + | + | + | - |
| OPEST099 | + | + | + | + | + |
| OPEST102b | + | + | + | + | - |
| OPEST106 | + | - | + | - | - |
| OPEST120b | + | + | + | + | - |
| OPEST126b | - | + | - | - | - |
| OPEST131 | + | + | - | + | + |
| OPEST156 | - | - | - | - | - |
| OPEST169 | + | + | + | - | - |
| OPEST177b | + | - | - | - | - |
| OPGSSR001 | - | + | - | - | - |

+ detected signal on capillary electrophoresis in both labs, - no signal detected or multiband profile.

**Table S2.** Error rate per allele and per locus for other species datasets.

**Table S3.** CC and delta values for each tested clustering method.

| **method** | **CC^a^** | ***delta* (0.5)** | ***delta* (1)** |
| --- | --- | --- | --- |
| nearest neighbour (NN) | 0.910 | 0.328 | 0.348 |
| furthest neighbour (FN) | 0.897 | 0.157 | 0.193 |
| weighted pair group (WPGMA) | 0.943 | 0.083 | 0.105 |
| unweighted pair group (UPGMA) | 0.946 | 0.081 | 0.101 |
| weighted pair-group centroid | 0.896 | 0.851 | 0.890 |
| unweighted pair-group centroid | 0.903 | 0.719 | 0.753 |
| Ward´s method | 0.478 | 0.742 | 0.770 |

^a^Cophenetic correlation coefficient.

**Table S4.** Identity analysis with 0 allelic mismatch allowed.

**Table S5.** Identity analysis with 1 allelic mismatch allowed.

**Table S6.** Identity analysis with 2 allelic mismatch allowed.

**Table S7.** Identity analysis with 3 allelic mismatch allowed.

**Table S8a.** Identity analysis with 4 allelic mismatch allowed.

**Table S8b.** Identity analysis with 4 allelic mismatch allowed.

**Table S9a.** Identity analysis with 5 allelic mismatch allowed.

**Table S9b.** Identity analysis with 5 allelic mismatch allowed.

**Table S10.** Empirical evaluation of 21 published SSR markers.

^a^number of alleles for all accessions together, ^b^number of alleles within *P. somniferum* only.


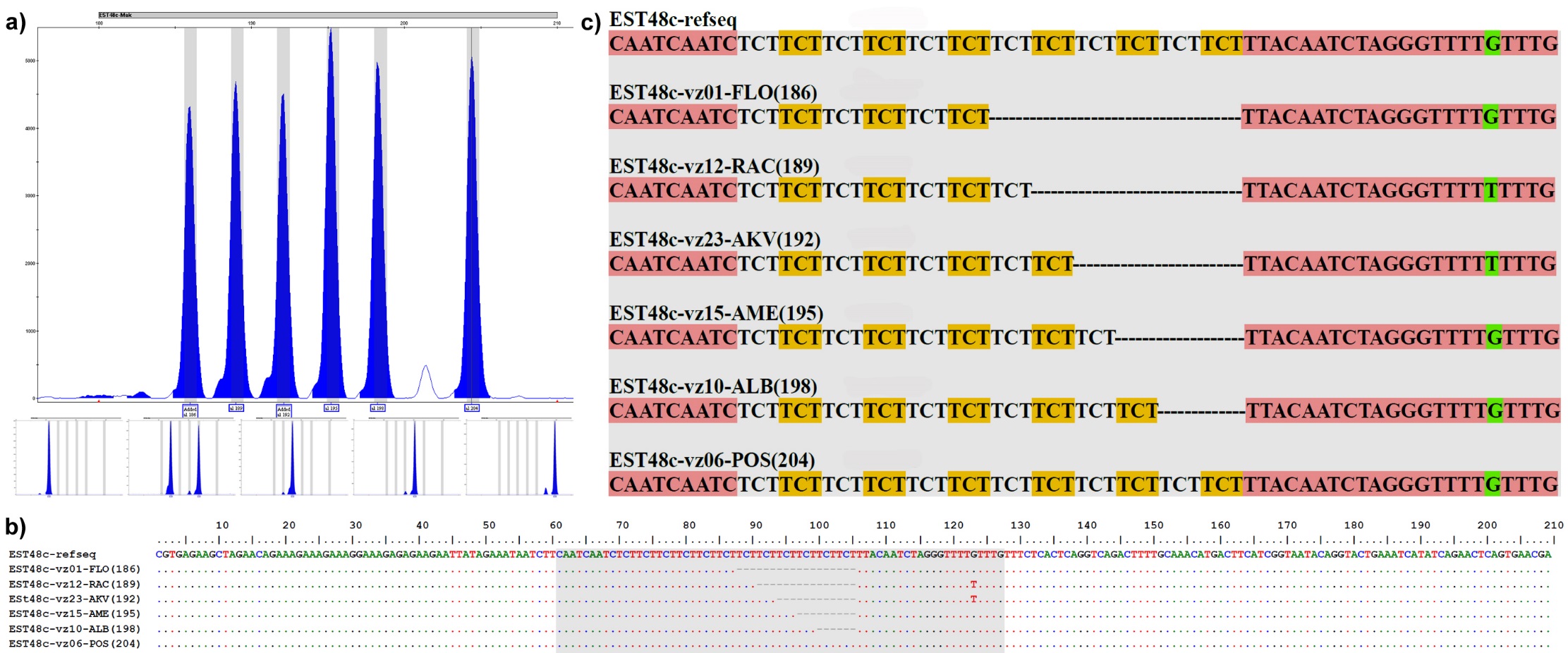


**Figure 1.** Example of allelic ladder for marker OPEST48c. Part a) shows whole allelic ladder resolved by capillary electrophoresis that contain alleles from individual genotypes (smaller pictures). Part b) shows defined alleles through sequences of individual genotypes, including reference sequence where microsatellite motif with short part of flanking regions are highlighted (grey zone) and zoomed (Part c). This is the older version of the ladder containing only alleles from culinary varieties of *P. somniferum*.
